# A Malignant Course of Anomalous Right Coronary Artery Arising From Left Coronary Cusp Presenting With Exertional Syncope

**DOI:** 10.7759/cureus.25922

**Published:** 2022-06-14

**Authors:** Manisha Raikar, Pushpa Khanal, Taimoor Haider, Deepakraj Gajanana

**Affiliations:** 1 Internal Medicine, Guthrie Robert Packer Hospital, Sayre, USA; 2 Cardiology, Arnot Ogden Medical Center, Elmira, USA

**Keywords:** anomalous course, interarterial course, ct chest angiogram, sudden cardiac death, congenital

## Abstract

Anomalous origin of the right coronary artery from the left sinus of Valsalva is a rare congenital disease. It is mostly benign, with malignant variants reported in a few instances. One such "malignant course" is its course between the main pulmonary artery and the aortic root. It is relatively uncommon but may present with angina or sudden cardiac death (SCD) in the absence of significant atherosclerosis, especially in young patients. Therefore, diagnosis becomes pivotal.

Here, we report a case of a female in her late 70s with a history of vertigo who presented to the hospital with exertional syncope without prodromal symptoms. Further workup demonstrated high-sensitivity troponin that peaked at 3300 ng/dl. She was evaluated by cardiology for NSTEMI (non-ST segment elevation myocardial infarction) and underwent a coronary angiogram that identified non-obstructive coronary artery disease but an anomalous origin of the right coronary artery arising from the left coronary cusp. She underwent a CT (computed tomography) chest angiogram, which demonstrated an interarterial course between the aorta and pulmonary artery with multiple areas of significant stenosis. After extensive discussion, she decided to be treated conservatively due to its benign condition and late presentation. Identification of this anomalous course becomes pivotal as surgical correction can improve patient outcomes.

Definitive therapy is surgery with unroofing of intramural segments, stenting, or surgical intervention with bypass grafting, reimplantation of the anomalous artery, or osteoplasty. However, in older patients, conservative management with exercise limitations is an acceptable option.

## Introduction

An anomalous coronary artery from the opposite sinus (ACAOS) is a rare entity with a reported incidence of 1.3% undergoing coronary arteriography at the Cleveland Clinic Foundation from 1960 to 1988 [1-3]. Often asymptomatic, it is an incidental finding during cardiac catheterization. 81% were benign anomalies, whereas the rest included ectopic origin from the pulmonary artery, ectopic origin from the opposite aortic sinus, single coronary artery, and large coronary fistulae with profound consequences [1-3]. This 19% contributes to one-third of sudden cardiac deaths (SCD) in young patients and is the second leading cause of sudden cardiac death in athletes after hypertrophic cardiomyopathy [4]. In an angiography study of 1,950 patients by Angelini et al., the incidence of anomalous coronary arteries was 5.6%. The incidence of the RCA arising from the left coronary cusp was 0.92% [5]. The anomalous right coronary artery (ARCA) was first described in 1948 by White and Edwards [6]. The ARCA is more prevalent than the anomalous left coronary artery (ALCA) and accounts for a majority of SCD [7-9]. Patients with ALCA are younger in age compared to ARCA [7-9]. Age at presentation is variable in patients with ARCA, as often they go undetected [10]. Mortality rates are higher in ALCA (57%) when compared to ARCA (25%) [10].

## Case presentation

A female in her late 70s with a history of hypertension, hyperlipidemia, and diabetes mellitus presented with one episode of traumatic syncope. Upon presentation, her heart rate was 78 bpm and her BP was 140/90 mm Hg. The remainder of her vital signs were unremarkable. A physical examination demonstrated a bruise over the left forehead without any obvious deformity. The physical examination demonstrated normal cardiovascular and respiratory system examination. A neurological examination demonstrated no obvious focal neurological deficits. The remainder of the physical examination was unremarkable. 

**Figure 1:**
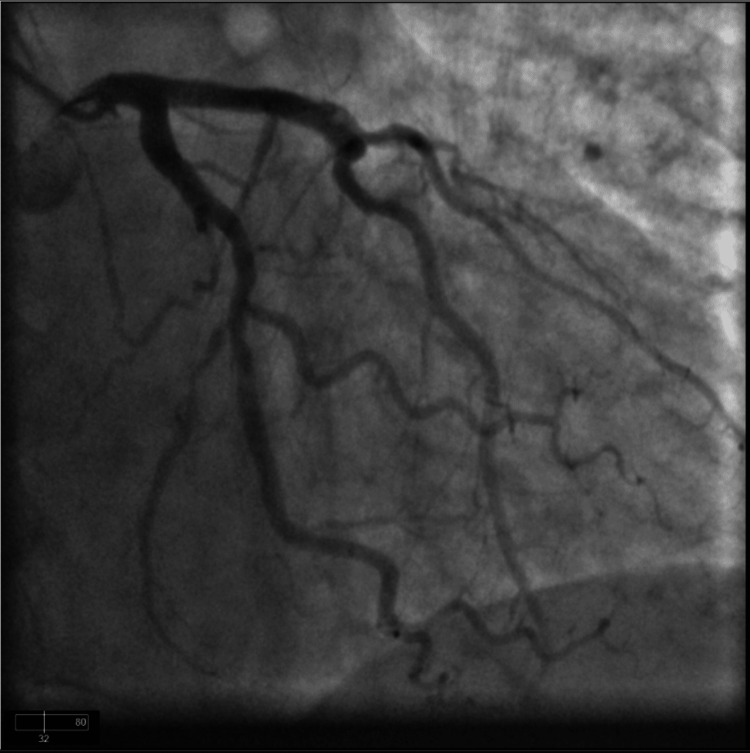
Coronary cineangiography demonstrating left anterior descending and left circumflex coronary artery originating from left coronary cusp.

**Figure 2:**
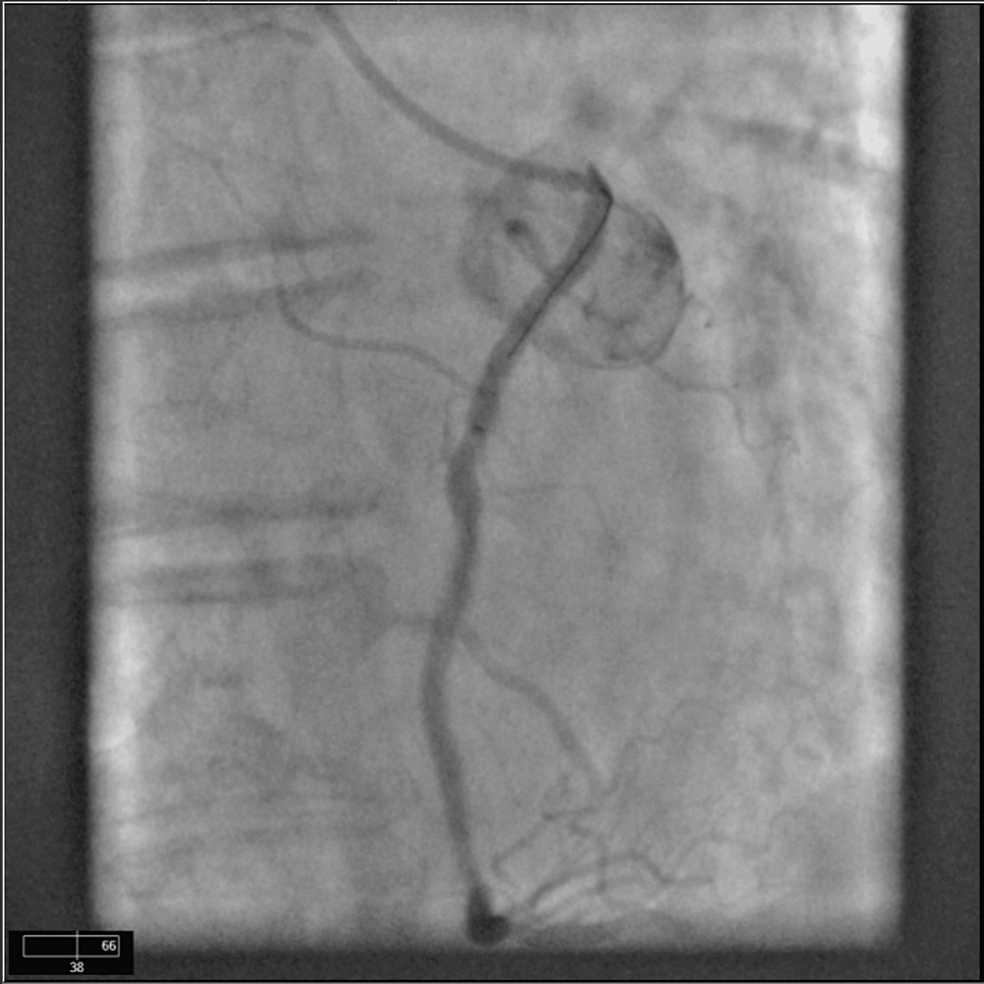
Coronary cineangiography demonstrating right coronary artery originating from left coronary cusp.

**Figure 3:**
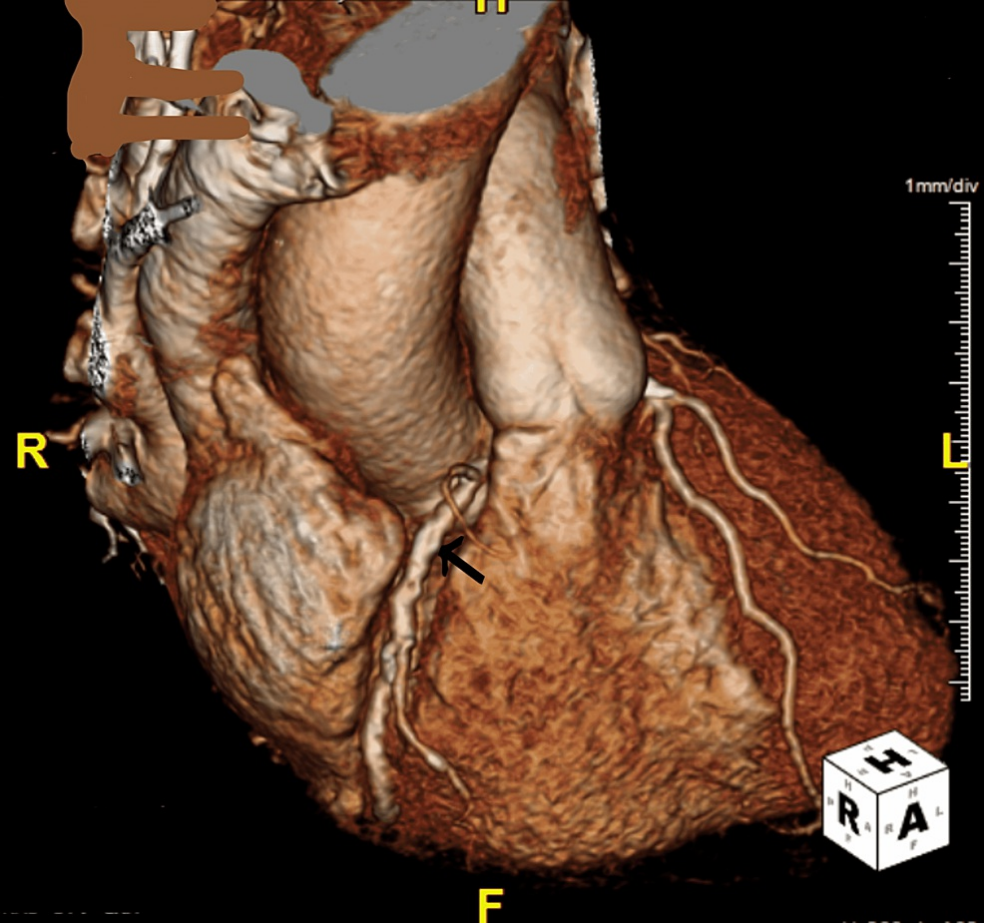
3D reconstruction image of coronary computerized tomography demonstrating anomalous right coronary artery arising from left coronary cusp and traversing between aorta and main pulmonary artery.

The baseline serum creatinine was 1.4 mg/dl and the GFR was 60 ml/min/1.73 m2. The high sensitivity troponin peaked at 3760 ng/dl and trended down to 3680 ng/dl. She was started on intravenous heparin infusion for non-ST elevation myocardial infarction. A 2D transthoracic echocardiogram revealed an ejection fraction of 55% without any wall motion abnormalities. There was mild aortic valve sclerosis without any other valvular abnormalities. After giving informed consent, she underwent cardiac catheterization. Her cardiac catheterization demonstrated no occlusive coronary artery disease. It was suggestive of an anomalous origin of the right coronary artery from the left coronary cusp (Figures 1-2). It was suspicious of the probable intraarterial course of the right coronary artery. She eventually underwent high-resolution coronary CT that demonstrated an anomalous right coronary artery from the left coronary cusp (Figure 3). It demonstrated an inter-arterial course between the main pulmonary artery and the ascending aorta.

## Discussion

A symptomatic anomalous coronary artery from the opposite sinus presents with symptoms similar to acute coronary syndrome [11]. In our particular case, the person who was diagnosed with ARCA presented with exertional syncope. ARCA is further classified based on its course into two categories: a high interarterial course (when ARCA travels between the aorta and pulmonary artery) and a low interarterial course (when ARCA travels between the aorta and right ventricular outflow tract). The reason for differentiation is important as the higher course is associated with major adverse cardiac events (MACEs) and most likely will need surgical intervention when compared to low course ARCA [11].

The pathophysiology based on current theories is transient ischemia. Proposed mechanisms are: (1) ostial stenosis due to acute take-off angle, slit-like orifice, and compression of the intramural segment by the aortic valve commissure [12]. (2) mechanical compression, which is contributed to adrenergic surge during exertion, leads to increased cardiac output resulting in expansion of the aorta and pulmonary artery, which leads to mechanical compression of the right coronary artery if it has a high interarterial course as seen in our patient; and (3) vasospasm of anomalous artery [13,14]. An intravascular ultrasound study [12] states that luminal compression of the coronary artery was totally attributed to the aorta because pulmonary artery pressure was lower than the aorta. Some autopsy-based studies state ostial stenosis is associated with sudden cardiac death [15,16]. This transient ischemia leads to malignant arrhythmias and sudden cardiac death [16]. The probable cause for late presentation in our patient is when the ischemic threshold had been surpassed due to the addition of coronary artery disease secondary to age.

Coronary computed tomography angiography (CCTA), also known as multidetector row helical CT (MDCT), and cardiac MRI are the most effective imaging studies to diagnose ACAOS [17,18]. In particular, MDCT is especially optimal due to its higher spatial resolution, which also helps to accurately distinguish patients as it provides multiplanar image reconstruction to evaluate the course of the artery [18]. High-risk features are very well demonstrated by mentioning specific CT-derived anatomical criteria that are associated with an increased risk for MACEs such as unstable angina and myocardial infarction [14].

Once recognized, the treatment options need to be carefully determined. In patients with asymptomatic or symptomatic ALCA, surgical repair is indicated due to its substantial risk of sudden cardiac death [10]. But in patients with ARCA, the treatment path is not clear as most cases are benign due to the lower risk of sudden cardiac deaths in these patients, especially if it is not a higher interarterial course [11]. One recommendation states surgical intervention in symptomatic young (<35 years) patients. Asymptomatic young patients are on a case-to-case basis and the degree of luminal narrowing and high-risk features [19,20]. Older patients should most preferably choose conservative management with exercise limitation due to its benign course and conservative treatment, unroofing of intramural segments, stenting or surgical intervention with bypass grafting, reimplantation of the anomalous artery, or osteoplasty [14,20]. Coronary angiography and angioplasty with stent placement are difficult due to small, slit-like orifices and long, curved intramural portions of the anomalous artery. Success rates of selective cannulation are 55-61%, which is also because of limited experience by interventional cardiologists due to its rarity [19,20]. MDCT-guided cannulation is more successful and therefore helpful in selective cannulation. The unroofing procedure enlarges the orifice, creates an acute angulation, and decreases lateral compression of the intramural segment. Complications involve aortic valve incompetence. The coronary artery bypass graft is easier, but the native artery is patent at rest, leading to competition flow, which can be overcome by ligation of the native anomalous artery [20]. Coronary reimplantation to the right coronary sinus is possible but has a risk of neo-ostial stenosis [20].

## Conclusions

Although anomalous coronary arteries from the opposite sinus are rare, they can lead to catastrophic outcomes like sudden cardiac death. The greatest challenge is to detect the abnormality accurately as physicians, especially cardiologists, are unaware of this disease due to its rarity and should keep this as a differential diagnosis. Routine testing with electrocardiography and echocardiography is not sensitive enough to diagnose congenital abnormalities and needs further investigation.
